# Multimodal deep learning model for enhanced early detection of aortic stenosis integrating ECG and chest x-ray with cooperative learning

**DOI:** 10.3389/fradi.2025.1698680

**Published:** 2025-11-25

**Authors:** Shun Nagai, Makoto Nishimori, Masakazu Shinohara, Hidekazu Tanaka, Hiromasa Otake

**Affiliations:** 1 Division of Cardiovascular Medicine, Department of Internal Medicine, Kobe University Graduate School of Medicine, Kobe, Japan; 2 Division of Molecular Epidemiology, Kobe University Graduate School of Medicine, Kobe, Japan

**Keywords:** multimodal AI, cooperative learning, aortic stenosis, deep learning, ECG, CXR=chest x-Ray

## Abstract

**Background:**

Aortic stenosis (AS) is diagnosed by echocardiography, the current gold standard, but examinations are often performed only after symptoms emerge, highlighting the need for earlier detection. Recently, artificial intelligence (AI)–based screening using non-invasive and widely available modalities such as electrocardiography (ECG) and chest x-ray(CXR) has gained increasing attention for valvular heart disease. However, single-modality approaches have inherent limitations, and in clinical practice, multimodality assessment is common. In this study, we developed a multimodal AI model integrating ECG and CXR within a cooperative learning framework to evaluate its utility for earlier detection of AS.

**Methods:**

We retrospectively analyzed 23,886 patient records from 7,483 patients who underwent ECG, CXR, and echocardiography. A multimodal model was developed by combining a 1D ResNet50–Transformer architecture for ECG data with an EfficientNet-based architecture for CXR. Cooperative learning was implemented using a loss function that allowed the ECG and CXR models to refine each other's predictions. We split the dataset into training, validation, and test sets, and performed 1,000 bootstrap iterations to assess model stability. AS was defined echocardiographically as peak velocity ≥2.5 m/s, mean pressure gradient ≥20 mmHg, or aortic valve area ≤1.5 cm^2^.

**Results:**

Among 7,483 patients, 608 (8.1%) were diagnosed with AS. The multimodal model achieved a test AUROC of 0.812 (95% CI: 0.792–0.832), outperforming the ECG model (0.775, 95% CI: 0.753–0.796) and the CXR model (0.755, 95% CI: 0.732–0.777). Visualization techniques (Grad-CAM, Transformer attention) highlighted distinct yet complementary features in AS patients.

**Conclusions:**

The multimodal AI model via cooperative learning outperformed single-modality methods in AS detection and may aid earlier diagnosis and reduce clinical burden.

## Introduction

Aortic stenosis (AS) is one of the most prevalent and severe valvular heart diseases, particularly in elderly populations. The number of patients is estimated at approximately 16.1 million across the 37 advanced economies, and the presence of many undiagnosed cases is regarded as an additional clinical challenge (1, 2). Echocardiography is the gold standard for diagnosing AS (3, 4), yet it demands specialized equipment and expertise, and many facilities face a shortage of trained personnel (5). Moreover, in numerous cases, echocardiographic evaluation occurs only after symptom onset, underscoring the need for earlier detection strategies (3, 4).

On the other hand, electrocardiogram (ECG) and chest x-ray (CXR) are inexpensive, non-invasive tests readily accessible in most healthcare settings. Recently, advances in artificial intelligence (AI) have led to the development of high-performing single-modality models using ECG or CXR to detect AS and other cardiac conditions (6–9). However, single-modality approaches have inherent limitations, and clinical decision-making usually integrates multiple diagnostic sources rather than relying solely on a single modality. Reflecting this reality, multimodal AI models have gained increasing attention in the medical domain (10). However, such multimodal AI approaches have rarely been applied to the diagnosis of valvular heart disease, including AS, combining ECG and CXR—two modalities with distinct yet complementary characteristics—holds promise for enhancing both diagnostic accuracy and early detection of AS.

Furthermore, traditional multimodal AI approaches typically employ either an early fusion or late fusion strategy, but these may not fully capture the intricate interactions between different data modalities. Hence, in this study, we introduce a “cooperative learning” framework, wherein the ECG model and CXR model iteratively refine one another's predictions throughout the training process. This approach preserves the unique features of each modality while harnessing their complementary strengths (11). To our knowledge, this is the first application of cooperative learning in the valvular heart disease domain, particularly for the diagnosis of AS, and we hypothesize that this multimodal deep learning strategy may surpass conventional models in enabling early and more accurate detection.

Therefore, the primary objective of this study is to develop and evaluate a novel multimodal AI model that integrates ECG and CXR data using cooperative learning to improve diagnostic performance and facilitate earlier diagnosis of AS. Additionally, we aim to compare this model's clinical utility against existing single-modality models to elucidate the benefits of a truly multimodal approach.

## Methods

### Overall study design

**Figure 1:**
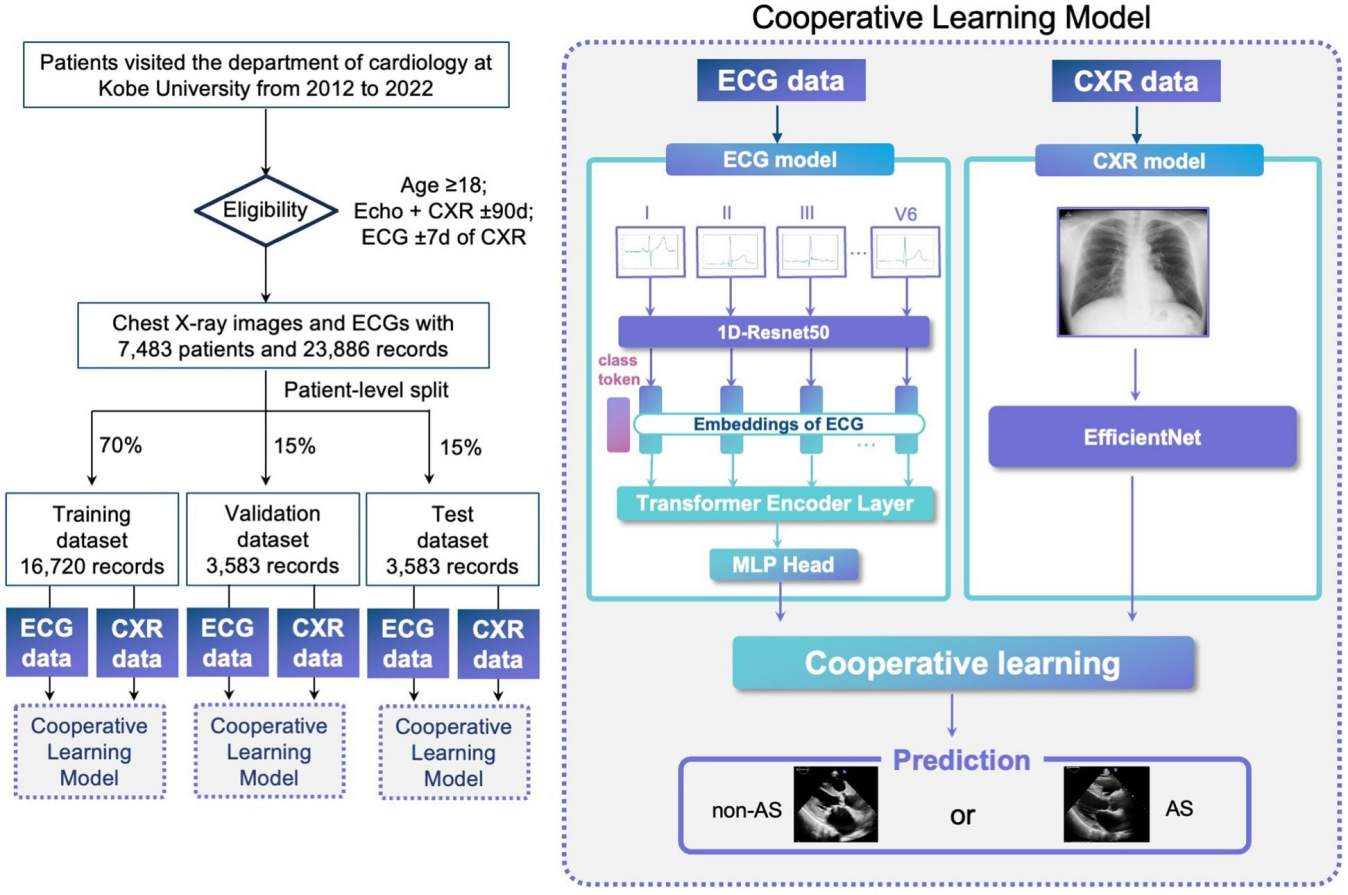
Overview of this study. Left: Cohort assembly and split. Adult patients (≥18 years) who visited the Department of Cardiology at Kobe University (2012–2022) and had chest x-ray (CXR) and echocardiography (Echo) within ±90 days plus a 12-lead ECG within ±7 days of the CXR were included. After linkage/de-duplication, 7,483 patients contributed 23,886 paired records, which were divided at the patient level into training/validation/test sets (70%/15%/15%; 16,720/3,583/3,583 records; no patient overlap). Right: Overview of the multimodal AI model for early detection of AS. The illustration summarizes the study design, data integration from ECG and CXR, cooperative learning framework, and model performance. AI, artificial intelligence; AS, aortic stenosis; CXR, chest x—ray; ECG, electrocardiogram.

The overall study design, including data acquisition, preprocessing, model training, validation, and testing, is outlined in Figure 1. In summary, a total of 23,886 records from 7,483 patients were collected, and the AI model was trained using ECG and CXR data to detect AS. The dataset was randomly split into training (70%), validation (15%), and test (15%) cohorts, with internal validation performed via bootstrap resampling (1,000 iterations). The model's performance was then compared against single-modality models to assess the advantage of a multimodal AI approach. Additionally, visualization techniques such as Grad-CAM and attention maps were applied to interpret model decisions and highlight key diagnostic features.

This study was approved by the Local Ethics Committee of our institution in conformity with the Declaration of Helsinki (No. B230191).

### Data sources

We utilized data from patients who visited the Department of Cardiology at Kobe University Hospital from January 2012 to November 2022. The inclusion criteria were patients aged 18 years or older who had undergone ECG, CXR, and echocardiography. For each patient, CXR images taken within three months before or after the echocardiography date were linked. If multiple CXR images were available within this timeframe, the image closest to the echocardiography date was selected. ECG data were matched to the CXR data using ECGs taken within one week before or after the date of CXR. The dataset included 23,886 patients. The ECG data were extracted in CSV format from 12-lead ECG devices with a sampling rate of 500 Hz, resolution of 1.25 µV, and a low-pass filter set at 150 Hz.

### Data preprocessing

**Figure 2:**
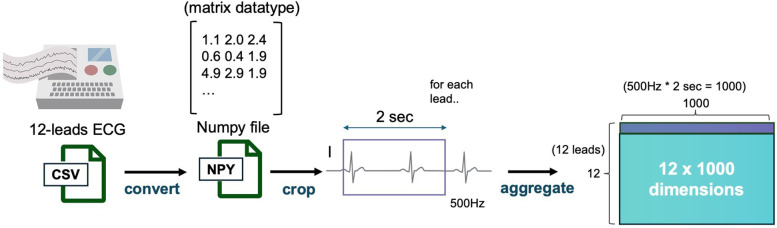
ECG data preprocessing pipeline. This figure depicts the data preprocessing steps applied to ECG data. ECG signals from 12 leads are sampled at 500 Hz, and a 2-second segment is extracted from each recording. The resulting data is converted into a 12 × 1,000 matrix and stored as a NumPy file for further model processing. ECG, electrocardiogram.

The dataset of 23,886 patient records was randomly split by patient ID into training, validation, and test datasets in a 70%, 15%, and 15% ratio, corresponding to 16,720, 3,583, and 3,583 records, respectively. Each patient had multiple sets of ECG and CXR data obtained on the same day. During training, for each iteration, a random 2 s segment was selected from the 15-second ECG recordings, resulting in a (12, 1,000) matrix for each sample, considering the 500 Hz sampling rate (Figure 2). The CXR data were extracted as DICOM files and converted to 8-bit images.

For data augmentation, the ECG data were calibrated by setting the baseline to 0, followed by the addition of random noise within a 0.1 mV range. The CXR images underwent augmentation involving random rotations of ±10 degrees and contrast adjustments with a 25% probability.

AS was defined as meeting at least one of the following criteria: aortic valve velocity ≥2.5 m/s, mean pressure gradient (mPG) ≥20 mmHg, or aortic valve area (AVA) ≤1.5 cm^2^ (12).

### Model construction

This study employed a multimodal model designed to simultaneously process ECG and CXR data.

### ECG model

The ECG data contain both local morphological features and long-term temporal dependencies.We processed the ECG data using a module that combines a one-dimensional ResNet50-based convolutional neural network (CNN) to extract local waveform features and a Transformer model to capture temporal relationships across multiple cardiac cycles (13–15). The CNN component was used to extract 256-dimensional feature vectors from each lead through a shared CNN model (ResNet50), embedding them into the same feature space. These vectors, together with a class token, were input into the Transformer encoder, which then output a vector corresponding to the class token, representing the integrated ECG features across all leads. This vector was then passed through a linear layer to generate the final prediction.The Transformer model was trained with a batch size of 32, using a learning rate that varied from 1 × 10^−3^ to 1 × 10^−7^ following a log-uniform distribution.The Adam optimizer was employed for the training process (16).

### CXR model

CXR data primarily contain spatial morphological information, which is best captured by convolutional neural network (CNN)-based architectures. Therefore, we employed an EfficientNet model pretrained on ImageNet, which has been widely used in medical image classification studies including chest radiography and has an established architecture (17, 18). The input images were resized to 224 × 224 pixels, and a sigmoid activation function was applied at the final layer.

### Cooperative learning model

We fused ECG and CXR through a cooperative learning framework (11), which minimizes a standard prediction error together with an agreement penalty between the two modalities. We minimize the population analogue of cooperative learning:

$$\underset{\left(f_{\mathrm{ecg}},f_{\mathrm{cxr}}\right)}{\min}\;E[1/2{\left(y-(f_{\mathrm{ecg}}(X_{\mathrm{ecg}})+f_{\mathrm{cxr}}(X_{\mathrm{cxr}}))\right)}^{2}+\rho /2{\left(f_{\mathrm{ecg}}(X_{\mathrm{ecg}})-f_{\mathrm{cxr}}(X_{\mathrm{cxr}})\right)}^{2}]$$

where $X_{\mathrm{ecg}}$ and $X_{\mathrm{cxr}}$ denote the ECG and CXR inputs for a single sample, y∈{0,1} is the binary label, $f_{\mathrm{ecg}}(\cdot )$ and $f_{\mathrm{cxr}}(\cdot )$ are neural networks with trainable parameters, $f_{\mathrm{ecg}}(X_{\mathrm{ecg}})$ and $f_{\mathrm{cxr}}(X_{\mathrm{cxr}})$ denote the scalar scores from the ECG and CXR branches, and ρ≥0 is a hyperparameter controlling the strength of the agreement term.

The first term (prediction term) fits the binary label using an additive model in which each branch contributes a score, so the optimizer adjusts the parameters of $f_{\mathrm{ecg}}\;\mathrm{and}\;f_{\mathrm{cxr}}$ so that their sum matches y. The second term (agreement term) acts as a co-regularizer that shrinks the difference $f_{\mathrm{ecg}}(X_{\mathrm{ecg}})-f_{\mathrm{cxr}}(X_{\mathrm{cxr}})$ toward zero per sample, encouraging both branches to capture the shared disease signal while damping modality-specific noise or artifacts.

The gradient w.r.t. $f_{\mathrm{ecg}}(X_{\mathrm{ecg}})$ is $-[y-(f_{\mathrm{ecg}}(X_{\mathrm{ecg}})+f_{\mathrm{cxr}}(X_{\mathrm{cxr}}))]+\rho (f_{\mathrm{ecg}}(X_{\mathrm{ecg}})-f_{\mathrm{cxr}}(X_{\mathrm{cxr}}))$ showing that the first term pulls the sum toward y and the second pulls the difference toward 0. Following the original framework, ρ=0 reduces to early fusion (joint fitting via the prediction term only), whereas ρ=1 particularly in the additive/linear setting—approaches late fusion by aligning the two marginal predictions. The hyperparameter ρ was determined using Bayesian optimization on the validation set. This formulation allows bidirectional feature alignment and flexible control between early and late fusion, while maintaining interpretability and stability during training.

### Training and hyperparameter selection

All models were implemented in PyTorch Lightning.

– Optimizer & precision. Adam optimizer (default *β*_1_ = 0.9, *β*_2_ = 0.999) with mixed-precision training (FP16).
– Scheduler. Cosine annealing (CosineAnnealingLR) with T_max_ = 10.
– Batch size. Tuned over {64, 128} via Bayesian optimization.
– Learning rate. Sampled from a log-uniform distribution [10^−6^, 10^−3^] via Bayesian optimization.
– Max epochs & early stopping. Up to 100 epochs with early stopping on validation loss (patience = 20). The checkpoint from the best validation loss was used for evaluation.
– Data augmentation for CXR data. Random rotation (−10° to +10°) and random autocontrast (probability = 0.25) during training.
– Model selection/objective for hyperparameter search. Hyperparameters—including ρ∈[0,1], batch size, and learning rate—were optimized by Bayesian optimization using validation AUROC as the objective metric. The final model reports results from the configuration that maximized validation AUROC, with weights taken from the epoch achieving the lowest validation loss under that configuration.

### Visualization of decision basis

The model combined a CNN and Transformer, and the visualization of decision basis involved a two-step process:

**Phase 1**: The attention layer from the Transformer model was extracted to identify which leads of ECG were being focused on. **Phase 2**: Using GradCAM, activation maps from the final CNN layer of the 1D-ResNet50 model were obtained (19). These maps were weighted according to the importance determined in Phase 1, resulting in a final heat map.

### Faithfulness ablation study

To quantitatively evaluate the causal relevance of the Grad-CAM highlights for the CXR classifier, we performed perturbation-based ablation (20–22). For deletion, we replaced the Grad-CAM top-p% pixels [p∈(18,30,40,50)] with a Gaussian blur applied in the model's normalization space. For insertion, we started from a heavily blurred image and progressively re-inserted the top-p% pixels from the original image. We used the positive-class logit as the score and summarized results by the area over the perturbation curve (AOPC). As baselines, we used area-matched random (pixels sampled uniformly at random) and a uniform map. Uncertainty was estimated with 1,000-sample bootstrap to obtain 95% CIs.

### Statistics

To evaluate the performance of the binary classification model in identifying AS, we used the following metrics: Recall, Precision, F1-Score, Accuracy, area under the curve—receiver operating characteristic (AUROC), and area under the curve—precision-recall (AUC-PR). We calculated 95% confidence intervals (CI) for these metrics and compared models using bootstrap methods, conducting two-tailed significance tests to determine *P*-values (23).

### Software

The base code was written in Python 3.10, utilizing PyTorch 2.0 for deep learning (24). The scikit-learn package was used for calculating evaluation metrics.

## Results

### Patient population and baseline characteristics

A total of 23,886 records from 7,483 patients were included in this study. They were randomly split into training (16,720 cases: 846 with AS, 15,874 without AS), validation (3,583 cases: 308 with AS, 3,275 without AS), and test (3,583 cases: 288 with AS, 3,295 without AS) datasets. The number of unique participants was 4,489 in the training set and 1,497 each in the validation and test sets. All patients were consecutively enrolled from a single institution, with AS diagnosis confirmed by standard echocardiographic assessment. Overall, 608 patients (8.1%) were diagnosed with AS. Detailed baseline characteristics are summarized in Table 1.

**Table 1:** Baseline characteristics of patients.

Variable	Overall (*N* = 7,483)	AS (*N* = 608)	Non-AS (*N* = 6,875)	*P*-value
Age, years	66 ± 16	79 ± 9.2	64 ± 15.9	<0.01
Gender (female), *n* (%)	2,948 (39.4)	336 (55.3)	2,612 (38.0)	<0.01
Body surface area, m^2^	1.61 ± 0.23	1.52 ± 0.19	1.62 ± 0.23	<0.01
Systolic blood pressure, mmHg	128 ± 28.4	133 ± 21.6	127 ± 29	<0.01
Heart rate, bpm	70.5 ± 18	69.6 ± 12	71 ± 18	0.23
Atrial fibrillation, *n* (%)	1,129 (15.0)	61 (10.0)	1,068 (15.6)	<0.01
LV end-diastolic volume, mL	86 ± 41.8	77.7 ± 35.7	86.7 ± 42.2	<0.01
LV end-systolic volume, mL	38.4 ± 31.7	31.9 ± 24.8	39 ± 32.2	<0.01
LVEF, %	59 ± 12.3	61.7 ± 11.9	58.8 ± 12.3	<0.01
LV stroke volume index, mL/m^2^	28.6 ± 11.7	30.3 ± 10.1	29.5 ± 10.6	0.15
Left atrial volume index, mL/m^2^	44.8 ± 25.2	56.7 ± 26.5	43.6 ± 24.8	<0.01
AV-Peak V, m/s	2.73 ± 1.35	3.72 ± 1.04	2.02 ± 1.09	<0.01
AV-mPG, mmHg	22.9 ± 21.3	35.6 ± 19.8	12.8 ± 16.1	<0.01
AVA, cm^2^	1.4 ± 0.83	0.94 ± 0.41	1.8 ± 0.88	<0.01

Baseline characteristics of the study population. Data are presented as mean ± SD or median (interquartile range) for continuous variables, and as *n* (%) for categorical variables. *P*-values indicate comparisons between patients with AS (*n* = 608) and those without AS (*n* = 6,875).

AS, aortic stenosis; AV, aortic valve; AVA, aortic valve area; LV, left ventricle; LVEF, left ventricular ejection fraction; V, velocity.

### Performance of the ECG model

We first developed a Single Transformer Classifier using only ECG data and performed 1,000 bootstrap iterations to validate its stability. On the test dataset, the median AUROC was 0.775 (95% CI: 0.753–0.796). Additional performance metrics, including sensitivity and specificity, are presented in Table 2.

**Table 2:** Performance metrics of single and multimodal models.

Model	AUROC	AUPRC	Accuracy	Precision	Recall	Specificity	F1-score
ECG Model	0.775 (0.753–0.796)	0.187 (0.161–0.218)	0.900 (0.893–0.907)	0.130 (0.113–0.161)	0.740 (0.635–0.794)	0.681 (0.635–0.798)	0.221 (0.196–0.256)
*p*-value	0.004	<0.001	<0.001	0.116	0.868	0.160	0.100
CXR Model	0.755 (0.732–0.777)	0.160 (0.134–0.187	0.938 (0.932–0.944)	0.121 (0.106–0.140)	0.755 (0.680–0.814)	0.051 (0.046–0.068)	0.209 (0.186–0.234)
*p*-value	<0.001	<0.001	<0.001	0.006	0.890	0.064	<0.001
Multimodal Model	0.812 (0.792–0.832)	0.300 (0.256–0.345)	0.859 (0.850–0.868)	0.160 (0.139–0.189)	0.752 (0.672–0.807)	0.741 (0.704–0.806)	0.263 (0.235–0.298)

Comparison of performance metrics (AUROC, AUPRC, Accuracy, Precision, Recall, Specificity, and F1-score) for single-modality (ECG or CXR) and multimodal models in detecting AS. Values are shown with 95% confidence intervals in parentheses. *P*-values indicate comparisons vs. the multimodal model.

AUROC, area under the receiver operating characteristic curve; AUPRC, area under the precision-recall curve; CXR, chest x—ray; ECG, electrocardiogram.

### Performance of the CXR model

Similarly, we trained a model using only CXR images and evaluated its performance with 1,000 bootstrap iterations. The median AUROC on the test set was 0.755 (95% CI: 0.732–0.777), as shown in Table 2.

### Performance of the cooperative learning multimodal model

**Figure 3:**
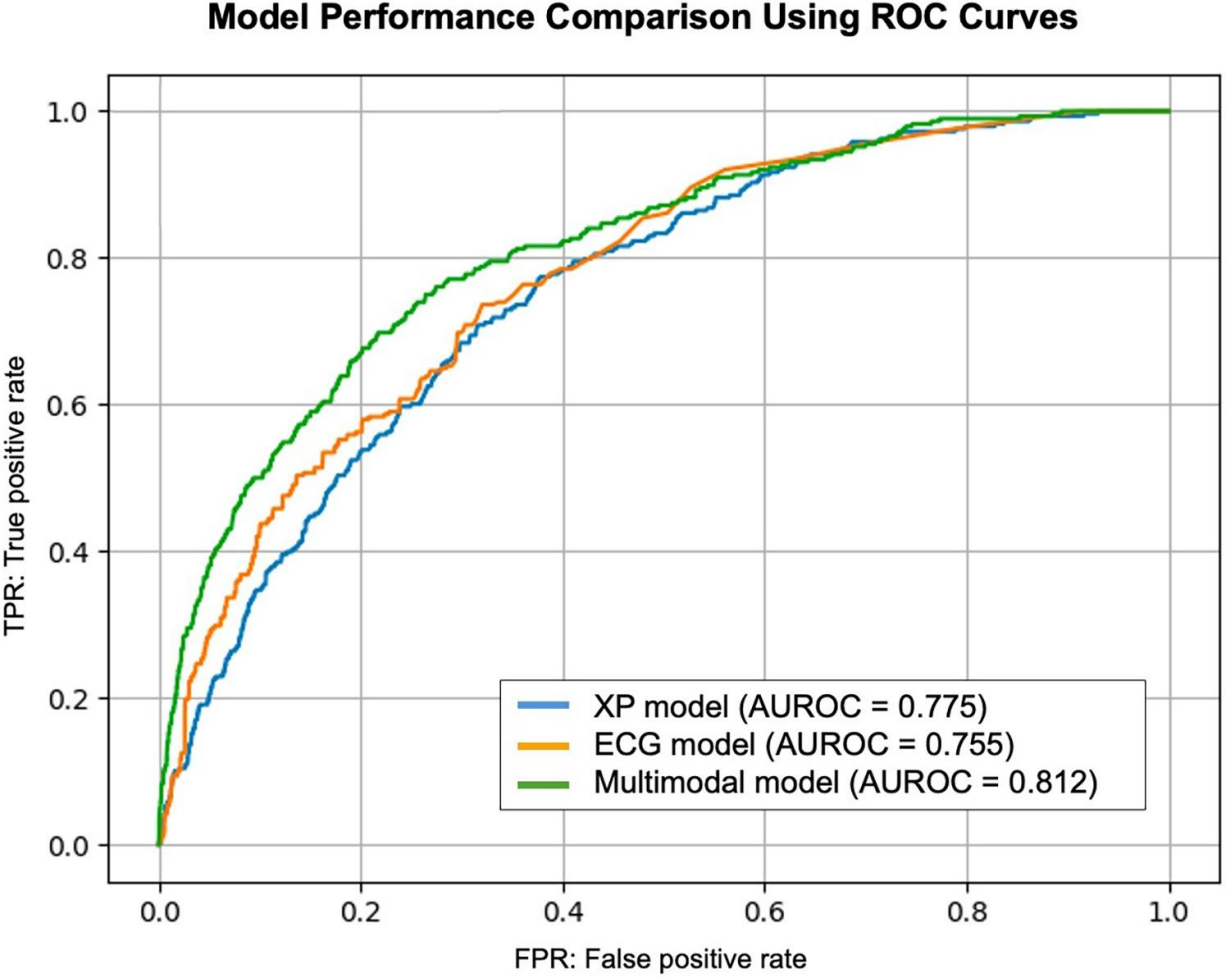
Model performance comparison using ROC curves. This figure presents the ROC curve for the models used in this study. The AUROC values indicate the classification performance for aortic stenosis detection. The cooperative learning multimodal model demonstrates superior performance compared to single-modality models. AUROC, area under the receiver operating characteristic curve; ROC, receiver operating characteristic.

For the cooperative learning model integrating both ECG and CXR data, 1,000 bootstrap iterations yielded a test AUROC of 0.8111, with a median of 0.812 (95% CI: 0.792–0.832). This significantly outperformed both single-modality models (*P* < 0.05, Table 2). Compared with the CXR model, the difference in AUROC showed a 95% CI of 0.032–0.080, and compared with the ECG model, the 95% CI was 0.015–0.058, both differences reaching statistical significance (*P* < 0.05, Figure 3).

In addition to AUROC, we also evaluated clinically relevant metrics—including sensitivity, specificity, and both positive and negative predictive values—all of which further supported the utility of the multimodal approach (Table 2).

### Model visualization

**Figure 4:**
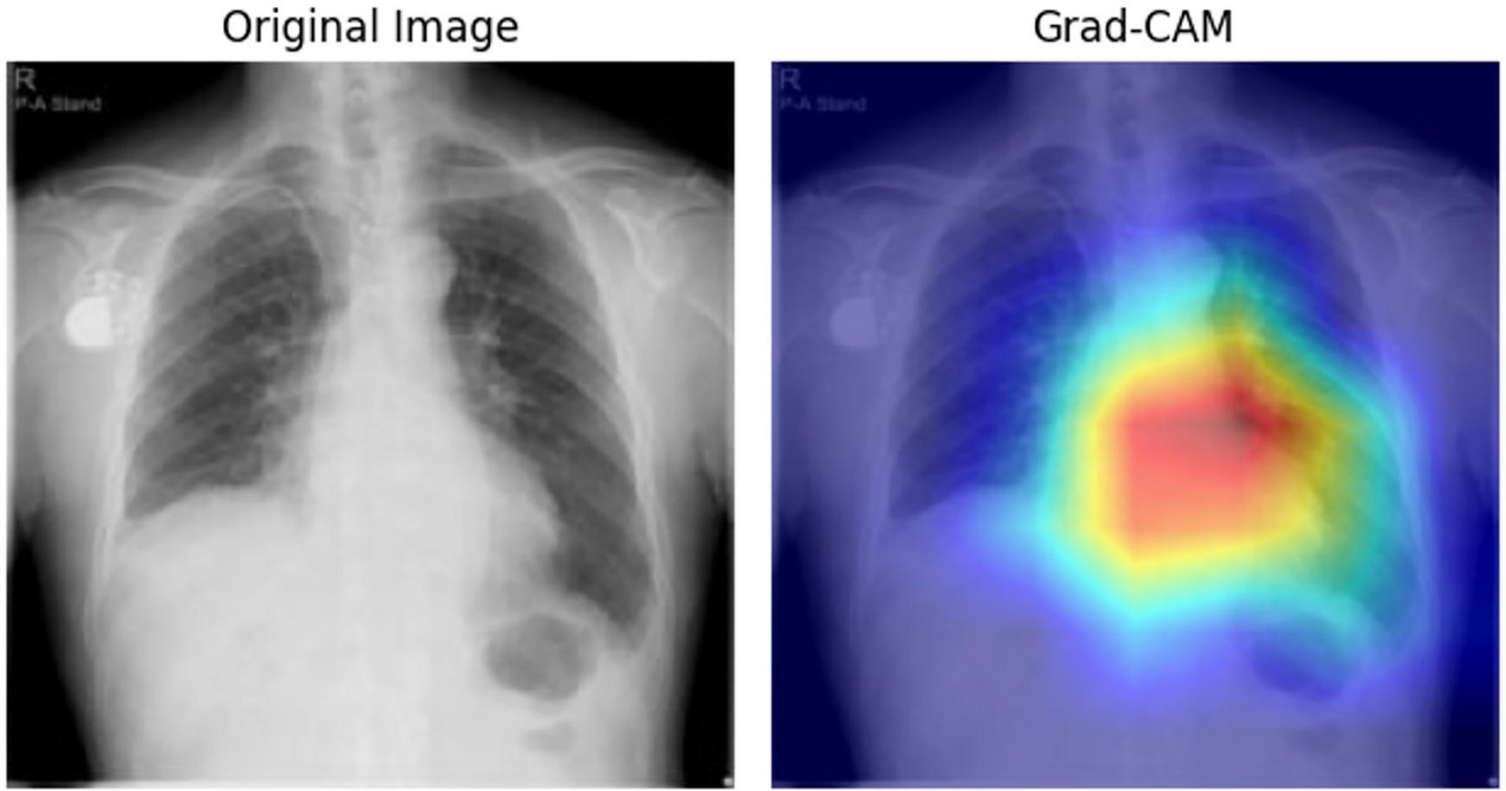
Grad-CAM visualization for CXR data. This figure illustrates the Grad-CAM visualization applied to CXR images for model interpretability. The visualization highlights the specific regions in the input image that contributed most to the model's classification of AS. Warmer colors in the heat map on the right indicate areas of higher relevance based on the model's internal features. AS, aortic stenosis; CXR, chest x—ray; Grad-CAM, gradient-weighted class activation mapping.

**Figure 5:**
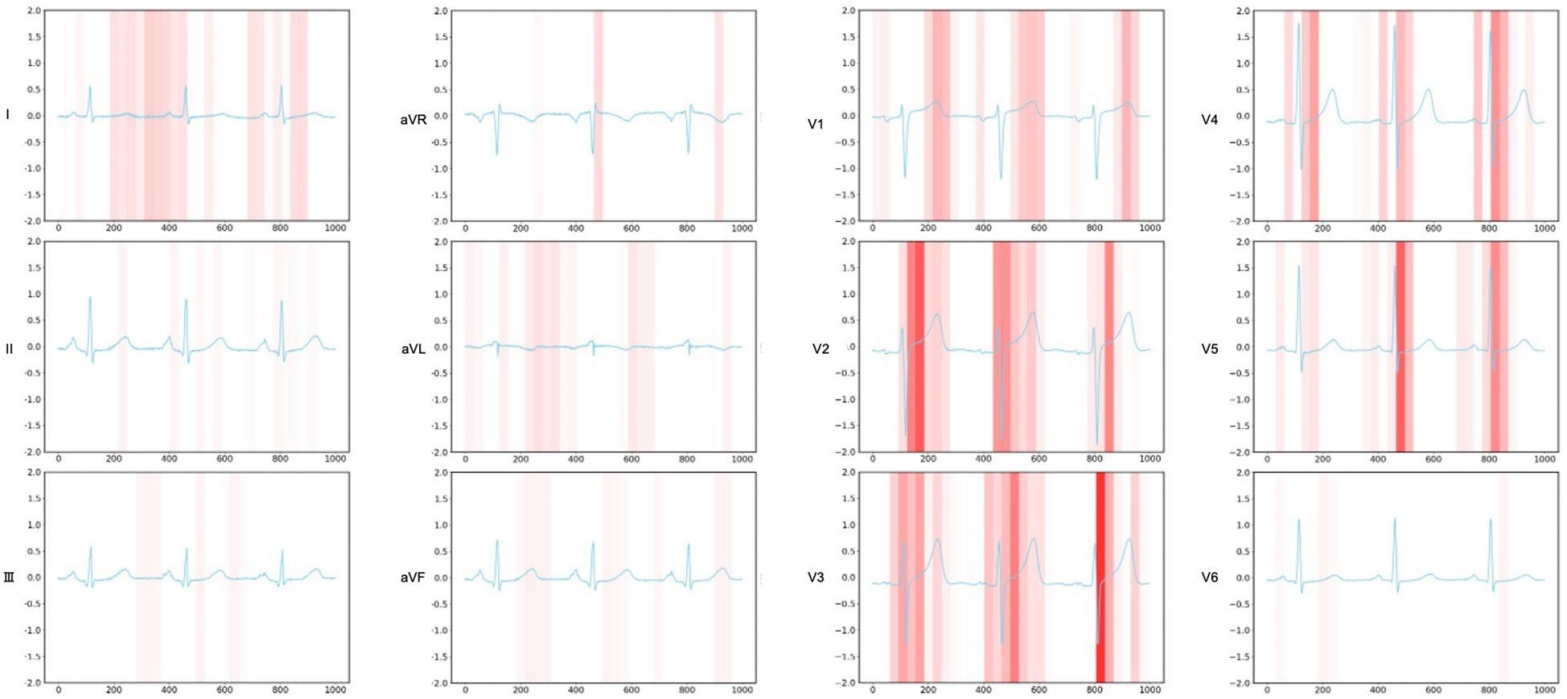
Attention mapping for ECG data. This figure presents the attention mapping results from the ECG model. The visualization demonstrates which specific leads and time segments in the input ECG signal were most influential in identifying patients with AS. Attention weights are displayed using a color gradient, with stronger signals indicating greater model focus. AS, aortic stenosis; ECG, electrocardiogram.

To elucidate the decision-making process, we applied Grad-CAM to the ResNet architecture for CXR images (Figure 4) and generated attention maps for the Transformer-based ECG model (Figure 5). These visualizations revealed characteristic regions of interest, such as cardiac silhouette enlargement on CXR or specific QRS wave patterns on ECG, which were highlighted in AS patients. Such findings suggest that the cooperative learning approach effectively integrates multimodal information to support its diagnostic decisions.

Grad-CAM-guided insertion yielded a higher AOPC than area-matched random (AOPC 1.807; 95% CI: 1.779–1.838 vs. 1.759; 95% CI: 1.729–1.791), indicating that highlighted regions contain information that drives the prediction. In the deletion test, Grad-CAM and random were comparable (AOPC 1.803; 95% CI: 1.775–1.834 vs. 1.846; 95% CI: 1.818–1.878), consistent with the model relying on distributed/global cues in CXR rather than a few highly localized pixels. The uniform baseline was nearly identical to Grad-CAM (AOPC ≈ 1.805–1.802) (Supplementary Figures S1A,B).

## Discussion

In this study, we demonstrated that combining a multimodal approach using ECG and CXR with a cooperative learning framework can significantly improve the early detection of AS. A total of 23,886 patient records from 7,483 patients were divided into training (70%), validation (15%), and test (15%) datasets, among which 608 patients (8.1%) were diagnosed with AS. Compared with single-modality models, the median AUROC of the cooperative learning model reached 0.812 (95% CI: 0.792–0.832), significantly outperforming the ECG model (0.775, 95% CI: 0.753–0.796) and the CXR model (0.755, 95% CI: 0.732–0.777; *P* < 0.05). This suggests that fusing multiple data modalities can enhance diagnostic performance beyond what either modality could achieve individually.

Recent studies have shown that not only severe AS but also moderate AS patients have a poor prognosis, highlighting the need for an earlier diagnostic framework (25). However, currently, echocardiography, which is the gold standard for diagnosing valvular diseases, faces challenges due to a shortage of skilled technicians and equipment, and AS is often evaluated only after symptoms appear. One proposed solution to this challenge is the application of AI in diagnostic technologies.

Several recent studies have reported AI-based severity assessment models using echocardiographic videos (13, 26). Furthermore, AI models that predict AS from a single modality, such as ECG or CXR images, have also been reported (7, 8). Among these reports, Ueda et al. developed an AI model using CXR images for detecting severe AS, and Pierre et al. reported an ECG-based model for detecting valvular diseases including severe AS. Both models demonstrated high diagnostic performance. However, in clinical practice, ECG interpretation can be affected by arrhythmias, making its evaluation unreliable.Similarly, patients with difficulties in maintaining posture or controlling respiration may yield unclear CXR images. Given these challenges, comprehensive assessment incorporating multiple modalities is commonly performed. AI-based diagnosis is also expected to follow this trend, with multimodal approaches becoming the mainstream rather than relying on a single modality. While some studies have reported AI-based multimodal models for predicting cardiac diseases (10, 27), there have been no reports specifically focusing on multimodal AI models for valvular disease diagnosis. Our study may represent a pioneering attempt in this field.

Furthermore, another key novelty of this study is the adoption of a cooperative learning framework, wherein ECG and CXR models exchange information in a complementary manner during training, distinguishing it from traditional early fusion and late fusion approaches. This method, known as cooperative learning, has been shown to achieve higher diagnostic accuracy compared to these conventional multimodal learning techniques, namely early fusion and late fusion (28). Although this method itself is not particularly new in the field of machine learning, this study represents its first application to valvular heart disease, specifically AS, and demonstrated that it can achieve our objectives with higher diagnostic accuracy.

In clinical practice, AS is known to cause electrical changes such as left ventricular hypertrophy and increased intracardiac pressure, which are reflected in ECG, as well as morphological changes such as left ventricular enlargement and aortic valve calcification, which manifest in CXR images. However, single-modality models may overlook subtle findings that could be detected only when multiple perspectives are integrated. The cooperative learning model allowed visualization through Grad-CAM and attention maps, revealing that both modalities focused on characteristic regions of interest. This suggests that the model effectively learned complementary relationships between ECG-derived electrical burden patterns and CXR-based morphological changes.

Furthermore, in addition to AUROC, we evaluated clinically relevant metrics including sensitivity, specificity, positive and negative predictive values, as well as AUPRC and accuracy. In particular, the accuracy of 0.859 (95% CI: 0.850–0.868) and recall of 0.752 (95% CI: 0.672–0.807) indicate that the model achieves a practically useful performance level for screening purposes, especially considering the relatively low prevalence of AS in our cohort. The multimodal model outperformed the single-modality models across these metrics, potentially reducing both misdiagnoses and missed cases. This statistically validated improvement is particularly important for early detection of AS. The precision and F1-score may appear modest; however, given the relatively low prevalence of AS (8.1%) in our cohort, within a screening framework, maintaining high sensitivity and negative predictive value is of greater clinical importance. However, subgroup analyses revealed that the model's performance declined somewhat in patients with pacemakers or severe chest wall deformities, where neither ECG nor CXR provided clear indications of AS. In such cases, additional imaging techniques such as echocardiography or CT would still be necessary.

In this study, we aimed to enable earlier detection by including moderate cases in patient selection from the outset. As a result, our multimodal model suggests the potential for highly accurate early detection.

To strengthen both feasibility and clinical relevance, future research should validate the model externally across diverse populations and investigate its broader applicability to other forms of valvular and myocardial disease.

## Limitation

This study has several limitations. First, the dataset was derived from a single medical institution (or a relatively homogeneous set of institutions), which may limit the generalizability of the findings to other geographic regions or ethnic groups. Large-scale, multicenter studies and validation in diverse healthcare settings are necessary to confirm the robustness of this approach. Future multicenter collaborative studies are planned to further evaluate the generalizability of our model. Additionally, only 8.1% of our cohort had AS, reflecting an imbalanced dataset that warrants further evaluation of class imbalance techniques such as oversampling or class weighting. Moreover, although we employed bootstrap analyses and data splitting to ensure the stability of our results, an independent external cohort was not available for validation. Future research should incorporate external datasets to provide more objective performance evaluation.

## Conclusion

Our results suggest that a multimodal model integrating ECG and CXR data, combined with cooperative learning, can achieve higher diagnostic accuracy for AS than single-modality approaches, thus offering an innovative avenue for non-invasive and cost-effective early screening. This framework could potentially streamline diagnostic workflows, reduce clinical burden, and promote earlier therapeutic intervention. Future directions include expanding this cooperative learning approach to larger, more diverse patient cohorts and exploring its applicability to other valvular diseases and cardiomyopathies. Through these efforts, AI-driven diagnostic models may become a valuable asset in the broader landscape of cardiovascular medicine.

## Data Availability

The data analyzed in this study is subject to the following licenses/restrictions: Patient-level data cannot be shared publicly because of institutional and ethical restrictions. Data access is regulated by the Ethics Committee of Kobe University Hospital. (IRB information: Kobe University Hospital Clinical & Translational Research Center, Reference number: No. B230191). Requests to access these datasets should be directed to Shun Nagai, alaics_twiworld@yahoo.co.jp.

## References

[1] De Sciscio P Brubert J De Sciscio M Serrani M Stasiak J Moggridge GD. Quantifying the shift toward transcatheter aortic valve replacement in low-risk patients: a meta-analysis. Circ Cardiovasc Qual Outcomes. (2017) 10(6):e003287. 10.1161/CIRCOUTCOMES.116.003287 28600455

[2] d'Arcy JL Coffey S Loudon MA Kennedy A Pearson-Stuttard J Birks J Large-scale community echocardiographic screening reveals a major burden of undiagnosed valvular heart disease in older people: the oxvalve population cohort study. Eur Heart J. (2016) 37(47):3515–22. 10.1093/eurheartj/ehw229 27354049 PMC5216199

[3] Otto CM Nishimura RA Bonow RO Carabello BA Erwin JP 3rd Gentile F 2020 ACC/AHA guideline for the management of patients with valvular heart disease: a report of the American college of cardiology/American heart association joint committee on clinical practice guidelines. Circulation. (2021) 143(5):e72–e227. 10.1161/CIR.0000000000000923 33332150

[4] Vahanian A Beyersdorf F Praz F Milojevic M Baldus S Bauersachs J 2021 ESC/EACTS guidelines for the management of valvular heart disease: developed by the task force for the management of valvular heart disease of the European society of cardiology (ESC) and the European association for cardio-thoracic surgery (EACTS). Rev Esp Cardiol (Engl Ed). (2022) 75(6):524. 10.1016/j.rec.2022.05.006 35636831

[5] Won D Walker J Horowitz R Bharadwaj S Carlton E Gabriel H. Sound the alarm: the sonographer shortage is echoing across healthcare. J Ultrasound Med. (2024) 43(7):1289–301. 10.1002/jum.16453 38534218

[6] Ueda D Matsumoto T Ehara S Yamamoto A Walston SL Ito A Artificial intelligence-based model to classify cardiac functions from chest radiographs: a multi-institutional, retrospective model development and validation study. Lancet Digit Health. (2023) 5(8):e525–e33. 10.1016/S2589-7500(23)00107-3 37422342

[7] Elias P Poterucha TJ Rajaram V Moller LM Rodriguez V Bhave S Deep learning electrocardiographic analysis for detection of left-sided valvular heart disease. J Am Coll Cardiol. (2022) 80(6):613–26. 10.1016/j.jacc.2022.05.029 35926935

[8] Ueda D Yamamoto A Ehara S Iwata S Abo K Walston SL Artificial intelligence-based detection of aortic stenosis from chest radiographs. Eur Heart J Digit Health. (2022) 3(1):20–8. 10.1093/ehjdh/ztab102 36713993 PMC9707887

[9] Sangha V Nargesi AA Dhingra LS Khunte A Mortazavi BJ Ribeiro AH Detection of left ventricular systolic dysfunction from electrocardiographic images. Circulation. (2023) 148(9):765–77. 10.1161/CIRCULATIONAHA.122.062646 37489538 PMC10982757

[10] Soto JT Weston Hughes J Sanchez PA Perez M Ouyang D Ashley EA. Multimodal deep learning enhances diagnostic precision in left ventricular hypertrophy. Eur Heart J Digit Health. (2022) 3(3):380–9. 10.1093/ehjdh/ztac033 36712167 PMC9707995

[11] Ding DY Li S Narasimhan B Tibshirani R. Cooperative learning for multiview analysis. Proc Natl Acad Sci USA. (2022) 119(38):e2202113119. 10.1073/pnas.2202113119 36095183 PMC9499553

[12] Baumgartner H Hung J Bermejo J Chambers JB Edvardsen T Goldstein S Recommendations on the echocardiographic assessment of aortic valve stenosis: a focused update from the European association of cardiovascular imaging and the American society of echocardiography. J Am Soc Echocardiogr. (2017) 30(4):372–92. 10.1016/j.echo.2017.02.009 28385280

[13] Ahmadi N Tsang MY Gu AN Tsang TSM Abolmaesumi P. Transformer-based spatio-temporal analysis for classification of aortic stenosis severity from echocardiography cine series. IEEE Trans Med Imaging. (2024) 43(1):366–76. 10.1109/TMI.2023.3305384 37581960

[14] He K Zhang X Ren S Sun J, editors. Deep residual learning for image recognition. In: Proceedings of the IEEE Conference on Computer Vision and Pattern Recognition. Las Vegas, NV: IEEE Computer Society (2016).

[15] Vaswani A. Attention is all you need. Adv Neural Inf Process Syst. (2017) 30:5998–6008. 10.48550/arXiv.1706.03762

[16] Kingma DP Ba J. Adam: a method for stochastic optimization. *arXiv preprint arXiv:14126980* (2014).

[17] Tan M Le Q, editors. Efficientnet: rethinking model scaling for convolutional neural networks. In: International Conference on Machine Learning. Long Beach, CA: PMLR (2019).

[18] Abedalla A Abdullah M Al-Ayyoub M Benkhelifa E. Chest x-ray pneumothorax segmentation using U-net with efficientnet and resnet architectures. PeerJ Comput Sci. (2021) 7:e607. 10.7717/peerj-cs.607 34307860 PMC8279140

[19] Selvaraju RR Cogswell M Das A Vedantam R Parikh D Batra D, editors. Grad-Cam: visual explanations from deep networks via gradient-based localization. In: Proceedings of the IEEE International Conference on Computer Vision. Venice: IEEE Computer Society (2017).

[20] Samek W Binder A Montavon G Lapuschkin S Müller K-R. Evaluating the visualization of what a deep neural network has learned. IEEE Trans Neural Netw Learn Syst. (2017) 28(11):2660–73. 10.1109/TNNLS.2016.2599820 27576267

[21] Fong RC Vedaldi A. Interpretable explanations of black boxes by meaningful perturbation. In: IEEE editor. Proceedings of the IEEE International Conference on Computer Vision. Piscataway, NJ: IEEE (2017).

[22] Petsiuk V Das A Saenko K. Rise: randomized input sampling for explanation of black-box models. *arXiv preprint arXiv:180607421*. (2018).

[23] Fawcett T. An Introduction to ROC analysis. Pattern Recognit Lett. (2006) 27(8):861–74. 10.1016/j.patrec.2005.10.010

[24] Paszke A Gross S Massa F Lerer A Bradbury J Chanan G Pytorch: an imperative style, high-performance deep learning library. Adv Neural Inf Process Syst. (2019) 32.

[25] Jacquemyn X Strom JB Strange G Playford D Stewart S Kutty S Moderate aortic valve stenosis is associated with increased mortality rate and lifetime loss: systematic review and meta-analysis of reconstructed time-to-event data of 409 680 patients. J Am Heart Assoc. (2024) 13(9):e033872. 10.1161/JAHA.123.033872 38700000 PMC11179918

[26] Holste G Oikonomou EK Mortazavi BJ Coppi A Faridi KF Miller EJ Severe aortic stenosis detection by deep learning applied to echocardiography. Eur Heart J. (2023) 44(43):4592–604. 10.1093/eurheartj/ehad456 37611002 PMC11004929

[27] Amal S Safarnejad L Omiye JA Ghanzouri I Cabot JH Ross EG. Use of multi-modal data and machine learning to improve cardiovascular disease care. Front Cardiovasc Med. (2022) 9:840262. 10.3389/fcvm.2022.840262 35571171 PMC9091962

[28] Li Y Li H Wu F Luo J. Semi-supervised learning improves the performance of cardiac event detection in echocardiography. Ultrasonics. (2023) 134:107058. 10.1016/j.ultras.2023.107058 37295222

