# Supplementary material for: Multimodal deep learning model for enhanced early detection of aortic stenosis integrating ECG and chest x-ray with cooperative learning
Source: Front Radiol. 2025 Nov 25;5:1698680. doi: 10.3389/fradi.2025.1698680 (PMC12685832; doi:10.3389/fradi.2025.1698680)
Supplement: Supplementary file 1 [file Supplementaryfile1.docx]

**Supplementary Figures**


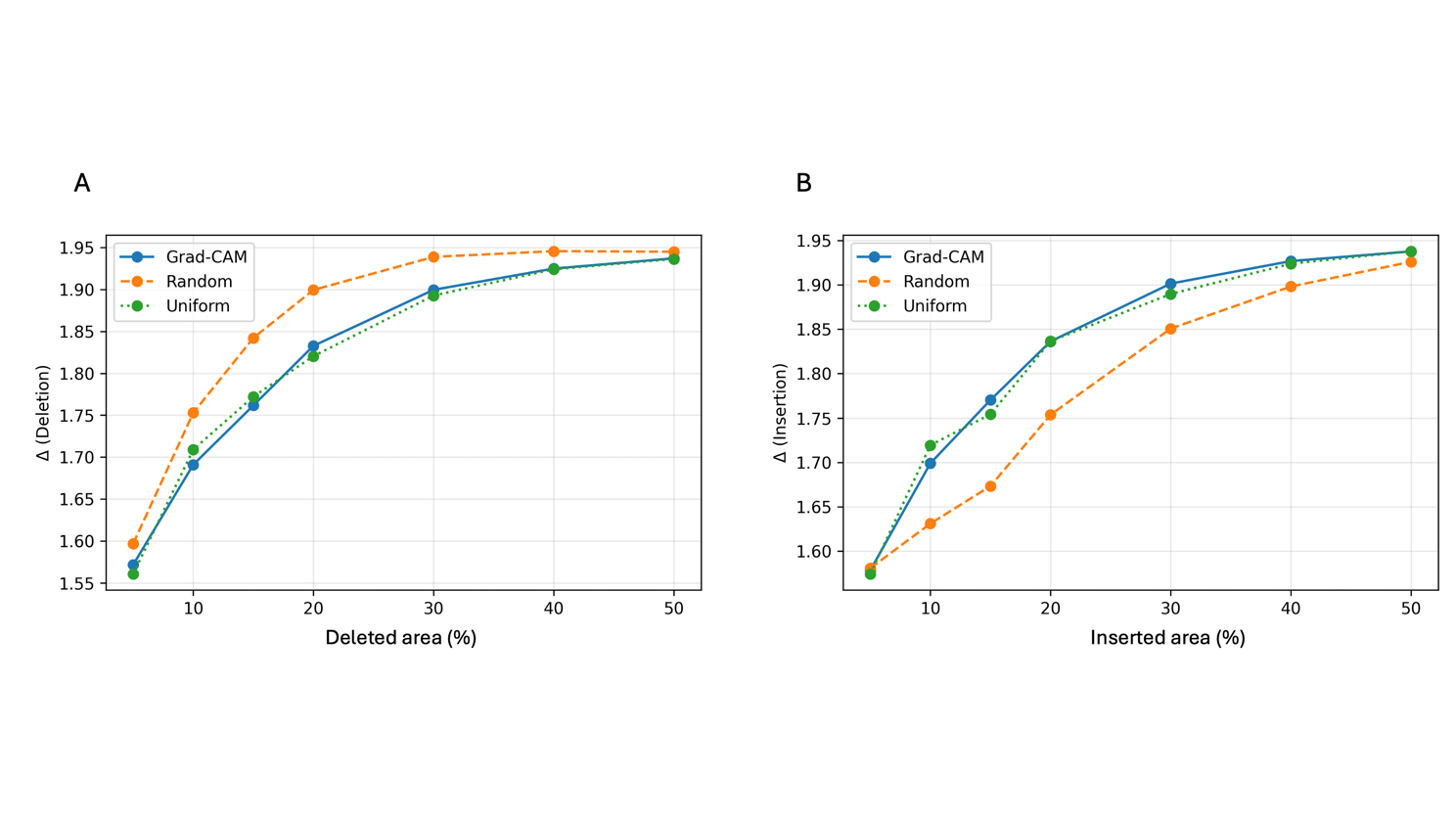


Supplementary Figure S1. Faithfulness ablation of the chest radiograph classifier (Deletion and Insertion).

(A) Deletion: mean Δ(logit) = f(x) − f(x_del) as a function of the deleted area p (%). (B) Insertion: mean Δ(logit) = f(x_ins) − f(x_base) as a function of the inserted area p (%). Lines denote Grad‑CAM (blue), Random (orange), and Uniform (green) maps; shaded bands indicate 95% bootstrap CIs across test images. AOPC (Deletion): Grad‑CAM 1.803 (95% CI 1.775–1.834), Random 1.846 (1.818–1.878), Uniform 1.802 (1.775–1.833). AOPC (Insertion): Grad‑CAM 1.807 (1.779–1.838), Random 1.759 (1.729–1.791), Uniform 1.805 (1.778–1.836). Scores are positive‑class logits; p∈{5,10,15,20,30,40,50}. Deletion used Gaussian blur in the normalization space; insertion started from a blurred base image. Area for Random and Uniform baselines was matched to Grad‑CAM at each p.
